# The role and intrinsic connection of cellular senescence and cell death in inflammatory bowel disease

**DOI:** 10.3389/fcell.2025.1502531

**Published:** 2025-04-24

**Authors:** Lichao Yang, Lianwen Yuan

**Affiliations:** Department of General Surgery, The Second Xiangya Hospital of Central South University, Changsha, China

**Keywords:** inflammatory bowel disease, cellular senescence, cell death, immunosenescence, inflammaging

## Abstract

Cellular senescence in the intestine can induce cell death, which extends beyond the mere clearance of senescent cells. This phenomenon is prevalent in inflammatory and immune-related diseases, particularly in inflammatory bowel disease (IBD). IBD is characterized by recurrent and chronic intestinal inflammation, with the occurrence and development of the disease being influenced by multiple factors, including genetics, environment, lifestyle, intestinal immunity, and gut microbiota. Chronic intestinal inflammation drives aging of the IBD immune system, reducing its efficiency and impairing the clearance of senescent cells. The disruption of cell death regulation and the interplay between cell death and cellular senescence contribute to disease progression in IBD, with inflammaging and immunosenescence playing the key role in this process. However, the mechanisms underlying the interplay between cell death and cellular senescence in the context of IBD remain unclear. Therefore, this paper comprehensively reviews the impact of cellular death and cellular senescence on intestinal aging in IBD, emphasizing the exploration of their potential interrelationships.

## 1 Introduction

Inflammatory bowel disease (IBD) is a recurrent and incurable inflammatory disorder of the gastrointestinal tract, primarily comprising Crohn’s disease (CD) and Ulcerative colitis (UC) (Plevris and Lees, 2022). The clinical course of IBD is unpredictable, characterized by recurrent abdominal pain, rectal bleeding, malnutrition, and a risk of malignancy. It is influenced by multiple factors, including genetic predisposition, dietary habits, environmental triggers, psychological factors, immune dysregulation, and gut microbiota imbalance (Bisgaard et al., 2022; Liu et al., 2022).

In recent years, as the standard of living has improved, the incidence of IBD has been rising globally, especially in developing countries, posing a significant threat to global health and healthcare systems (Aniwan et al., 2022; Agrawal et al., 2022). IBD exhibits clinical features similar to intestinal aging, such as intestinal dysfunction, malabsorption, and impaired barrier function. The persistent accumulation of senescent cells and limited clearance together drive the worsening of the inflammatory microenvironment in IBD (Sangfuang et al., 2025). Cell death serves as a key mechanism for clearing senescent cells in the body, with multiple pathways interwoven to maintain the dynamic balance of intestinal cell self-renewal (Ogrodnik et al., 2024). In IBD, cellular senescence and cell death collectively drive the pathological processes of the disease (Chavan et al., 2017). To date, the interactions between cellular senescence and cell death in IBD are not fully elucidated. In this review, we discuss the latest research developments on cellular senescence and cell death, with a particular emphasis on their roles and connections in IBD.

## 2 Cellular senescence in IBD

### 2.1 Immunosenescence in IBD

Immunosenescence refers to the gradual decline in immune system function with aging or prolonged inflammatory stimulation, involving extensive changes in both adaptive and innate immunity (Liu Z. et al., 2023). Recent studies have shown that this process is not only observed in the elderly population but is also commonly present in IBD patients, manifesting as “accelerated immunosenescence.” Chronic inflammatory environments induce premature immune cell senescence, impairing immune regulatory functions and exacerbating disease progression (Meng et al., 2023). In IBD, immunosenescence primarily manifests as a decline in T cell function, chronic low-grade inflammation, compromised mucosal immune barriers, and dysbiosis, all of which may affect disease progression and treatment outcomes (Yan et al., 2025; Kosinsky et al., 2024).

Studies have found that in IBD patients, the expression of KLRG1 and CD57 on CD4^+^ and CD8^+^ T cells increases, while CD28 expression decreases, indicating T cell exhaustion, with reduced proliferation ability and immune regulatory function (Li H. et al., 2025; Strunk, 2024; Bottois et al., 2020). Additionally, regulatory T cells in CD4^+^ T cells show impaired function, with reduced IL-10 production, weakening immune tolerance and inflammation suppression, leading to excessive activation of the immune system (Meng et al., 2023; Xie et al., 2022). In IBD patients, T cell telomere shortening and upregulation of senescence-related genes (*CDKN1A* and *CDKN2A*) also indicate that immune cells are entering a senescent state, further aggravating chronic inflammation (Hong and Katz, 2021). Immunosenescence affects the antigen presentation capacity of mucosal dendritic cells, leading to decreased immune tolerance to the microbiota in IBD patients, as evidenced by reduced probiotics and increased pathogenic bacteria, further accelerating immunosenescence (Meng et al., 2023; Wahida et al., 2021). In our previous research, single-cell RNA sequencing data from IBD patients showed an increase in the proportion of senescent CD8+T cells (KLRG1+CD28^−^) and a decrease in memory T cells (CD27^+^ CD28^+^), suggesting T cell exhaustion in IBD patients (Yao et al., 2024). Meanwhile, macrophages exhibited an inflammatory phenotype (M1 type), with high expression of IL-6 and TNF-α, indicating that chronic inflammation promotes the occurrence of immunosenescence in IBD (Yao et al., 2024).

Immunosenescence is also associated with reduced response to anti-TNF-α biologics (such as infliximab) in IBD patients, with mechanisms involving B cell dysfunction, T cell exhaustion, and the effects of the chronic inflammatory environment (Faye et al., 2024). In elderly IBD patients, the proportion of senescent B cells (CD21^−^CD11c+) increases, while memory B cells (CD27+IgD−) decrease, potentially leading to abnormal anti-infliximab antibody production and affecting drug efficacy (Meng et al., 2023; Childs et al., 2023). Additionally, T cell senescence (particularly the dysfunction of follicular helper T cells) may weaken the regulatory effect on B cells, further contributing to drug resistance (Di Micco et al., 2021; Varricchi et al., 2020). Meanwhile, the chronic inflammatory environment (with high expression of IL-6 and TNF-α) may accelerate the metabolism and clearance of infliximab, reducing its therapeutic effect (Ben-Horin et al., 2014). Therefore, immunosenescence not only impacts the disease progression of IBD but may also impair the long-term efficacy of biologics, highlighting the need for more precise personalized treatment strategies in the future.

### 2.2 Inflammation-induced senescence in IBD

IBD is characterized by chronic, recurrent intestinal inflammation, with its inflammatory microenvironment filled with pro-inflammatory cytokines (IL-6, TNF-α, IL-1β) and accompanied by extensive immune cell infiltration (Onyiah and Colgan, 2016). These inflammatory factors not only cause damage to intestinal epithelial cells but also promote cellular senescence through the activation of inflammatory pathways such as p38-MAPK, JAK-STAT, and NF-κB (Sienkiewicz et al., 2023). Studies have shown that in the IBD intestine, IL-6 can upregulate the expression of CDKN1A and CDKN2A through the JAK-STAT axis, leading to cell cycle arrest and inducing senescence (Yao et al., 2014). Additionally, TNF-α can directly trigger DNA damage response (DDR), accelerate telomere shortening, and further promote cellular senescence (Maekawa et al., 2018; Zhang et al., 2016).

The senescence-associated secretory phenotype (SASP) is an important link between cellular senescence and chronic inflammation. In IBD, senescent cells release large amounts of pro-inflammatory factors through SASP, including IL-6, IL-8, CCL2 (monocyte chemoattractant protein), and MMPs, forming a vicious cycle of inflammation-senescence (Almasabi et al., 2021). The inflammatory effect induced by SASP is mainly reflected in the fact that inflammatory factors, including IL-6 and IL-8, promote further immune cell infiltration, amplify the inflammatory response, and accelerate IBD-associated senescence (Bassotti et al., 2023). MMPs (matrix metalloproteinases) degrade the extracellular matrix (ECM), weakening the intestinal barrier, increasing intestinal permeability, and making IBD more prone to relapse (Lee and Kim, 2022). SASP promotes macrophages to maintain the M1 pro-inflammatory phenotype while suppressing the anti-inflammatory function of M2 macrophages, preventing the resolution of inflammation (Wang et al., 2024).

Intestinal stem cells (ISCs) play a key role in maintaining intestinal epithelial homeostasis. However, in the IBD inflammatory environment, ISCs are continuously stimulated by pro-inflammatory factors, which may lead to their senescence (Wang X. et al., 2020). Research has shown that Lgr5+ stem cells in IBD patients exhibit limited proliferation in the chronic inflammatory environment and show a senescent phenotype (Birch and Gil, 2020; Gorgoulis et al., 2019). Moreover, inflammation-induced mitochondrial dysfunction and abnormalities in the Wnt signaling pathway further affect the regenerative capacity of stem cells (Yun et al., 2023; Watanabe et al., 2022; Risques et al., 2008). SASP-released IL-6 and IL-1β can activate the JAK-STAT signaling pathway, upregulating the expression of CDKN1A and CDKN2A, and accelerating stem cell senescence (Wang X. et al., 2020; Yun et al., 2023).

Furthermore, IBD recurrence is closely related to the accumulation of senescent cells. When IBD transitions from remission to active phase, senescent cells can release pro-inflammatory factors through SASP, further enhancing the inflammatory microenvironment, making intestinal inflammation more likely to relapse (Birch and Gil, 2020; Gorgoulis et al., 2019).

## 3 Cell death in IBD

The human intestinal tract serves the vital functions of digesting food, absorbing nutrients and water, while also maintaining the balance of the gut microbiota. This places a considerable demand on intestinal epithelial cells, with approximately 10^10^ intestinal epithelial cells shedding and undergoing passive cell death every day (Blander, 2016). The passive shedding of intestinal epithelial cells primarily occurs at the tips of villi, with apoptosis being the primary mechanism of shedding (Iwanaga and Takahashi-Iwanaga, 2022). Recently, researchers have found that shed IECs can survive for several hours in mice, stimulating the expression of antimicrobial genes at the tips of villi and contributing to the regulation of gut microbiota composition (Bahar Halpern et al., 2023). This suggests that cell apoptosis can occur after IEC shedding. Moreover, compared to the small intestine, shed cells survive longer in the colon (Bahar Halpern et al., 2023),indicating potential differences in clearance mechanisms between the small intestine and colon, possibly beyond apoptotic clearance alone. The shedding and renewal of IECs are not only related to the proliferation of stem cells in the crypts but also to the clearance rate of senescent epithelial monolayer cells (Blander, 2016). In IBD, continuous shedding of IECs is observed, along with ongoing shedding of various immune cells, particularly more pronounced during active disease phases. While apoptosis is the normal physiological mechanism for cellular renewal in the intestine, other forms of cell death also play important roles in the development of IBD. In IBD, chronic inflammation and recurrent episodes lead to excessive cell death within the intestinal tract. This excessive cell death can activate the intestinal immune system, exacerbating inflammation in the IBD intestine (Günther et al., 2013; Dagenais et al., 2014). Multiple cell death mechanisms interact in the intestinal tract of IBD, cooperatively promoting disease progression.

In addition to apoptosis, several other regulated cell death pathways mentioned earlier are involved in the development of IBD. Different modes of cell death can affect the repair and regeneration of intestinal mucosal tissue, ultimately impacting intestinal fibrosis and even increasing the long-term risk of intestinal malignancies (Patankar and Becker, 2020). Excessive apoptosis has been shown to worsen intestinal inflammation in mice and IBD patients (Zhang J. et al., 2022), while anti-apoptotic strategies can help maintain colonic epithelial cell homeostasis and reinforce epithelial barrier function (Zhang et al., 2022b). Cell pyroptosis in the IBD intestine originates from the activation of inflammasomes (such as NLRP3) (Chen et al., 2019), which are integral to innate immune responses and play crucial roles in maintaining gut microbiota and gut-brain homeostasis (Man, 2018). Cell pyroptosis mediates various damage signals, leading to persistent chronic inflammation in IBD, primarily executed by proteins like GSDMB, GADMD, and GSDME (Zhang S. et al., 2022). Furthermore, GSDMB, an executor of pyroptosis, is also a critical factor in maintaining epithelial barrier function and inflammation resolution in IBD (Rana et al., 2022).

As detection methods continue to evolve, it has been discovered that cell death induced by trace metal elements also contributes to the development of intestinal inflammation in IBD. Ferroptosis, mediated mainly by endoplasmic reticulum stress and the NF-κB pathway, can be observed in intestinal epithelial cells of IBD patients and DSS mice (Ocansey et al., 2023). There is an accumulation of ROS, increased ferrous iron levels, and excessive lipid peroxidation in the intestinal epithelium in IBD, collectively driving chronic abnormal inflammation (Ocansey et al., 2023; Chen et al., 2020). Inhibitors of ferroptosis have been shown to effectively control chronic inflammation in the intestine, a fact widely validated in IBD patients and animal models (Arab et al., 2021; Xu et al., 2021; Huang et al., 2022; Zhu et al., 2022). Similarly, cuproptosis has also been observed in the intestine of IBD patients and DSS mice (Chen et al., 2022; Liu L. et al., 2023). Cuproptosis in the IBD intestine primarily affects the composition of immune cell infiltration and indirectly influences intestinal inflammation by controlling mitochondrial metabolism (Liu L. et al., 2023; Yang et al., 2023). While *SLC7A11*-driven disulfidptosis has not been extensively studied in IBD, the upregulation of the key gene *SLC7A11* in the intestinal mucosa of IBD is clear (Al-Mustanjid et al., 2020). Interestingly, SLC7A11 is an inhibitor of ferroptosis (Koppula et al., 2021), and ferroptosis promotes the occurrence and development of IBD intestinal inflammation (Wang et al., 2023). Therefore, it remains to be explored whether the high expression of SLC7A11 in the intestinal mucosa of IBD acts as a driver of disulfidptosis or a protector against ferroptosis. This warrants further in-depth research. To this end, our research team analyzed multiple UC datasets to identify differentially expressed genes associated with disulfidptosis and utilized lasso regression to determine key target genes. Immune infiltration analysis and the construction of clinical diagnostic models showed that five disulfidptosis-related immune-associated genes (*PDLIM1, SLC7A11, MYH10, NUBPL, OXSM*) are highly correlated with immune cells and inflammatory pathways in UC (Yang et al., 2024).

Lysoptosis-induced lysosomal membrane dysfunction (lysosome-dependent cell death) mediates cell death and further promotes the release of pro-inflammatory mediators, increasing the risk of colitis (Lassen et al., 2016; Wang et al., 2018). In IBD, ICD is a stress-driven form of RCD that can induce adaptive immune responses through antigen presentation and the release of danger signals (Kroemer et al., 2022). In the early stages of the disease, ICD-induced immune responses may play a regulatory role in tissue repair, helping to clear damaged cells and promote intestinal epithelial regeneration (Clucas and Meier, 2023). However, as the natural course of IBD progresses, the immune activation potential of ICD may have adverse effects in the context of chronic inflammation, such as exacerbating tissue damage or promoting carcinogenesis (Hayashi et al., 2021; Ruan et al., 2020).

Necroptosis in IBD mainly occurs in intestinal epithelial cells, and inhibiting RIPK3 can alleviate chronic intestinal inflammation induced by Necroptosis to some extent (Günther et al., 2011; Welz et al., 2011). Necroptosis can also occur in intestinal stem cells in IBD, and the deletion of the key gene *SETDB1* can induce Necroptosis, affecting colonic epithelial differentiation, disrupting mucosal barrier, and promoting intestinal inflammation (Wang R. et al., 2020; Južnić et al., 2021). Unlike regulated cell death, necrosis is not commonly observed in IBD and is less involved in the regulation of chronic enteritis. It generally occurs in acute mucosal injury or extreme external stimuli (Zhou et al., 2022).

## 4 Correlation between cellular senescence and cell death in IBD

### 4.1 Cellular senescence to cell death

In the intestinal tissues of IBD patients, intestinal epithelial cells, fibroblasts, immune cells, and others often exhibit clear signs of senescence (Wei et al., 2023; Giunta et al., 2022). Senescent cells typically face three possible fates: clearance by immune cells; maintenance in a quiescent state; or entry into regulated programmed cell death (such as pyroptosis, necrosis, autophagy, or apoptosis) (Fantini et al., 2024). However, under IBD conditions, due to impaired immune clearance, a large number of senescent cells fail to be removed in time, persist for extended periods, and exacerbate the negative effects of SASP. Persistent inflammatory stimuli can induce the activation of various regulated cell death (RCD) pathways, leading to further damage to the intestinal barrier (Tower, 2015). Specifically, senescent cells release a large number of pro-inflammatory factors through SASP and activate the NF-κB and p38 MAPK pathways, enhancing inflammatory signaling, making adjacent cells more susceptible to damage and even entering Necroptosis (Zhang et al., 2022d; Bertheloot et al., 2021). The accumulation of senescent cells may amplify the TNF-α-dependent RIPK3 overexpression, thereby exacerbating intestinal epithelial programmed necrosis (Necroptosis), and the release of damage-associated molecular patterns (DAMPs) from necrotic cells will further activate NF-κB signaling, intensifying the inflammatory response and the vicious cycle of cellular senescence (Hou et al., 2024; Royce et al., 2019). Additionally, senescence leads to increased intracellular oxidative stress, such as the activation of the NOX (NADPH oxidase) family, resulting in the accumulation of reactive oxygen species (ROS) that further promote NLRP3-Caspase-1 axis-mediated pyroptosis (Zarrin et al., 2021; Yuan and Ofengeim, 2024; Hsu et al., 2021). Cytokines secreted by SASP also inhibit AMPK activity, reducing ULK1-dependent autophagy initiation, thereby impairing the autophagic clearance function of intestinal cells (Hsu et al., 2021; Cheng et al., 2024).

**FIGURE 1:**
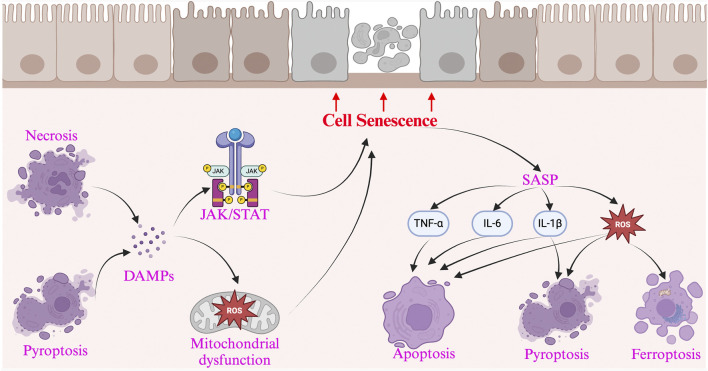
The interaction between cellular senescence and cell death in Inflammatory Bowel Disease and presents the key pathways and mechanisms involved. Cellular senescence promotes cell death through the SASP, which releases pro-apoptotic factors (such as TNF-α, IL-6, and IL-1β), triggering the Fas/FasL axis and Caspase cascade, leading to apoptosis. Additionally, SASP induces oxidative stress (ROS accumulation), activating the p53/p21 axis, accelerating DNA damage, and promoting cell death. It also activates the NLRP3 inflammasome via IL-1β and ATP, triggering pyroptosis, causing cell rupture and the release of DAMPs. On the other hand, cell death exacerbates cellular senescence by releasing DAMPs (such as HMGB1 and ATP), which continuously activate NF-κB, promoting chronic inflammation and inducing more cells into a senescent state. The inflammatory microenvironment activates the JAK/STAT axis, while excessive ROS accumulation leads to mitochondrial damage, accelerating DNA breaks, and further promoting the accumulation of senescent cells.

The accumulation of senescent cells in the intestine leads to aging-related changes, while persistent intestinal aging can reduce the expression of apoptosis-related genes in mesenchymal stem cells in IBD, affecting mucosal repair and regeneration (Alt et al., 2012). Consistent with reduced apoptosis in the state of intestinal aging, biomarkers of apoptosis in normal human serum decrease with age (Kavathia et al., 2009). Apoptosis is associated with the healing of mucosal ulcers in the intestinal tract (Wang et al., 2025). The downregulation of apoptosis gene expression in intestinal epithelial cells due to a decreased clearance rate of senescent cells may be a key factor contributing to the prolonged non-healing of intestinal ulcers in IBD (Li W. et al., 2025). In the animal models (such as the DSS-induced rat colitis model), the expression of SASP-related molecules (such as IL-1β, MMPs) is significantly elevated, accompanied by a reduction in mitochondrial membrane potential and an increased apoptosis rate in intestinal epithelial cells, further demonstrating the role of SASP in inducing cell death (Moiseeva et al., 2023). The main crosstalk mechanisms between cell death and cellular senescence in IBD are summarized in Figure 1.

In summary, senescent cells in the IBD intestine usually accumulate due to persistent inflammatory stimuli, oxidative stress, telomere shortening, and DNA damage, exhibiting resistance to apoptosis. It is important to note that senescent cells that inhibit apoptosis may promote malignant transformation, increasing the risk of IBD patients developing colorectal cancer (Schmitt et al., 2022; Lv et al., 2024).

### 4.2 Cell death to cellular senescence

In the progression of IBD, cell death is not merely an endpoint but may serve as a trigger for cellular senescence. Various regulated cell death modes, such as pyroptosis, apoptosis, necroptosis, and autophagy, promote the entry of intestinal epithelial cells into a senescent state through different mechanisms (Tower, 2015).

In the early stages of IBD, intestinal epithelial cells undergo excessive apoptosis in response to external stimuli, which may deplete the intestinal stem cell pool and force the stem cells into a senescent state (Kavathia et al., 2009). In the later stages of IBD, prolonged chronic inflammation may suppress apoptosis in intestinal epithelial cells, leading to a decreased clearance rate of senescent cells (Alt et al., 2012). Some cells that fail to undergo apoptosis may become arrested in the G1 phase, displaying typical features of senescence. In IBD, the highly activated pyroptosis in intestinal epithelial cells induces the release of IL-1β, IL-18, and DAMPs, which continuously activate the NF-κB pathway, creating a chronic inflammatory environment that promotes the senescence of neighboring cells (Chen et al., 2019). Senescent cells after pyroptosis also continue to secrete SASP, leading to mitochondrial dysfunction and exacerbating IBD-related cellular senescence. Furthermore, mutations in ATG16L1 and NOD2 in IBD reduce autophagic activity, making it difficult for senescent cells in the intestine to be cleared. Necroptotic cells also induce immune cell activation of the NF-κB pathway, promoting the accumulation of senescent cells (Wang R. et al., 2020). Cell death induced by metal elements is closely related to mitochondria (Tsvetkov et al., 2022; Jiang et al., 2021), as mitochondrial dysfunction mediates insufficient cellular energy supply, closely linking it to cellular senescence (Fantini et al., 2024). In conclusion, understanding the impact of different cell death modes on cellular senescence not only helps to uncover the pathological mechanisms of IBD but may also provide new intervention strategies for future IBD treatments. The impact of various cell death mechanisms on cellular senescence and their potential mechanisms in IBD are summarized in Table 1.

**TABLE 1:** The impact of various cell death mechanisms on cellular senescence and their potential mechanisms in IBD.

Cell death pathway	Classification	The impact on cellular senescence	Potential mechanism	References
Regulated cell death	Apoptosis	Inhibit	Apoptosis is a programmed cell death process that typically maintains tissue stability by eliminating cells that are severely damaged and irreparable. By removing these potentially harmful cells, apoptosis helps prevent the accumulation of senescent cells, thereby inhibiting the progression of senescence	Kurosaka et al. (2003), Elmore (2007), Majno and Joris (1995), Häcker (2000)
	Pyroptosis	Promote	Pyroptosis promotes cell senescence by triggering inflammation, which can lead to the accumulation of senescent cells and accelerate the aging process	Miao et al. (2023), You et al. (2023), Zychlinsky et al. (1992)
	Ferroptosis	Promote	Ferroptosis is an iron-dependent mode of cell death, typically accompanied by oxidative stress. Oxidative stress damages cells and may promote the onset of senescence	Dixon et al. (2012), Tang et al. (2021)
	Cuproptosis	Promote	Cuproptosis is a form of cell death triggered by the binding of copper ions to mitochondrial proteins, potentially leading to mitochondrial dysfunction and oxidative damage, thereby promoting senescence	Tsvetkov et al. (2022), Tang et al. (2022)
	Disulfidptosis	Promote	Disulfidptosis is caused by the rupture of disulfide bonds, typically accompanied by oxidative stress and protein dysfunction, which may promote the aging process	Zheng et al. (2023), Liu et al. (2023c)
	Necroptosis	Promote	Necroptosis is a cell death mode dependent on RIPK3 and MLKL proteins and is often associated with inflammatory responses. A persistent inflammatory environment may exacerbate cellular senescence, as inflammation promotes the release of senescence-associated cytokines	Seo et al. (2021), Degterev et al. (2005)
	Immunogenic Cell Death	Promote/Inhibit	Immunogenic cell death typically activates the immune system to recognize and clear damaged cells. In some cases, immune activation may lead to the clearance of more senescent cells, thus inhibiting senescence; however, excessive immune responses may promote chronic inflammation, exacerbating senescence	Galluzzi et al. (2018), Galluzzi et al. (2020)
Unregulated cell death	Necrosis	Promote	Necrosis is usually caused by severe cell damage, accompanied by the release of a large amount of cellular contents, which triggers a strong inflammatory response, promoting senescence	Holler et al. (2000), Vanlangenakker et al. (2012)

## 5 Conclusion and prospects

In IBD, chronic intestinal inflammation creates a pathological microenvironment that accelerates immune system aging and impairs its ability to clear senescent cells efficiently. The accumulation of senescent cells further disrupts tissue regeneration, leading to a complex interplay between cell death and cellular renewal. SASP and senescent cells modulate cell death pathways, but when the balance is lost due to chronic inflammation, cell death dysregulation can hinder senescent cell clearance, exacerbating disease progression.

Given the increasing focus on anti-aging therapies, targeting intestinal aging in IBD may provide a novel therapeutic strategy. However, research on drugs specifically targeting intestinal aging in IBD remains limited. Further basic and clinical studies are needed to explore whether preventing early intestinal aging in young IBD patients can mitigate symptoms and promote long-term remission. Although cell death regulation has been widely studied in IBD and some therapeutic interventions show promise, the complexity of IBD pathogenesis suggests that focusing solely on cell death mechanisms may not fully explain disease progression or treatment efficacy. Understanding the intrinsic link between cell death and cellular senescence is crucial for identifying new therapeutic targets, offering potential clinical and societal benefits in managing IBD.
